# Psoas Sarcopaenia Outcomes in Elderly Patients After Acute Lower Gastrointestinal Bleeding

**DOI:** 10.7759/cureus.74491

**Published:** 2024-11-26

**Authors:** Brian Cunningham, Megan McConnell, Aisling Daly, Paul Rice, Kevin McElvanna, Jane Kilkenny

**Affiliations:** 1 General Surgery, Northern Ireland Medical and Dental Training Agency, Belfast, GBR; 2 School of Surgery, Northern Ireland Medical and Dental Training Agency, Belfast, GBR; 3 School of Radiology, Northern Ireland Medical and Dental Training Agency, Belfast, GBR; 4 Radiology, Craigavon Area Hospital, Craigavon, GBR; 5 Colorectal Surgery, Craigavon Area Hospital, Portadown, GBR; 6 General Surgery, Royal Victoria Hospital, Belfast, GBR

**Keywords:** colorectal, frailty, nutrition, per-rectal bleeding, rectal bleeding, sarcopaenia

## Abstract

Introduction

Sarcopaenia has been examined as a predictor of frailty in surgical patients and may predict mortality. The hypothesis of this study is that sarcopaenia is associated with an increased risk of death following an episode of acute lower gastrointestinal bleeding (ALGB).

Methods

This study included patients admitted with ALGB between January 2017 and June 2022, who underwent cross-sectional imaging (CSI). CSI was accessed via imaging platforms, and the total psoas area (TPA) was calculated at the third lumbar vertebra (L3) by a radiology resident (post-Fellowship of the Royal College of Radiologists (FRCR)) and validated by a consultant radiologist. Clinical, mortality, and demographic data were obtained from the Northern Ireland Electronic Care Record (NIECR). Psoas muscle index (PMI) was calculated using TPA, standardising for height in mm^2^/m^2^. Sarcopaenia was defined as PMI <545 mm^2^/m^2^ for males and <385 mm^2^/m^2 ^for females.

Results

A total of 103 patients were included. Of these, 32 patients were defined as sarcopaenic (male, n = 20; female, n = 12). There was a statistically significant increase in mortality in the sarcopaenic group (Chi square (1, N = 103 = 7.582, p = 0.005888), odds ratio (OR) of 3.34, 95% confidence interval (CI) of 1.41-7.91). The Kaplan-Meier curve showed a significant decrease in survival probability in combined male and female groups (p < 0.001). There was a statistically significant association between sarcopaenic patients and length of hospital stay (LOHS) compared with non-sarcopaenic patients (p < 0.001).

Conclusions

Sarcopaenia as a predictor of frailty is an important risk factor for all-cause mortality in ALGB. CSI provides an opportunity to identify as well as investigate the aetiology of ALGB. Although radiation risk may limit its use, it should be considered when available.

## Introduction

With advances in healthcare, population age in both the developing and developed world is increasing [1]. The surgical population is ageing faster than the general population (1.8 v 0.8 years per annum), and those undergoing surgical procedures are on average 14.5 years older than the mean age of the population [2]. Acute lower gastrointestinal bleeding (ALGB) is an extremely common emergency general surgical condition. ALGB is associated with a wide range of aetiologies including haemorrhoids, diverticular bleeding, colitis, and malignancy [3]. It is estimated that there are approximately 85,000 cases of ALGB per annum in the United Kingdom (UK), with an incidence of 20 to 33 admissions per 100,000 adults per year [4,5].

Age is often used as an assumption of the level of fitness for surgery, endoscopy, and follow-up [6]. However, given the complexity of medical and surgical conditions, alongside individual patient characteristics, their chronological and physiological age are rarely equivalent [7]. Therefore, frailty has become an important factor in surgical decision-making. Various metrics are used to predict frailty. The most used metrics in the perioperative period are the American Society of Anaesthesiology (ASA) score, Physiological Operative Severity Score for the enUmeration of Mortality and Morbidity (POSSUM) score, and the National Emergency Laparotomy Audit (NELA) score [8]. The Rockwood Frailty Index, which is a component of the comprehensive geriatric assessment (CGA) is a commonly used frailty scoring system for non-operative admissions in the UK [9].

A key determinant of physical frailty is sarcopaenia. Sarcopaenia is a syndrome characterised by a progressive and generalised loss of skeletal muscle mass and strength. The number of people >60 years of age with sarcopaenia is estimated to be 1.2 billion worldwide and is expected to rise to 2 billion by 2050 [10]. Sarcopaenia is associated with an increased risk of mortality in a vast array of pathologies ranging from cardiovascular disease to cancer [11]. Computerised tomography (CT) and magnetic resonance imaging (MRI) are considered the gold standard method for identifying sarcopaenia. For the diagnosis of sarcopaenia, several metrics are used to evaluate muscle mass. These include the psoas muscle index (PMI), skeletal muscle index (SMI), and pectoralis muscle index [12]. In this study, we use the PMI at the level of the third lumbar vertebra (L3) that has been measured using CT imaging.

In surgical populations, sarcopaenia has been studied mainly through the lens of post-operative complications and mortality [13]. However, there has been little research to study the effect of sarcopaenia on outcomes after a hospital admission for a condition that is generally managed conservatively. To do this, we have analysed the effect of sarcopaenia after an episode of ALGB using two-year mortality data, length of hospital admission, discharge destination, and albumin levels.

## Materials and methods

Study design and patient selection

This was a dual-centre (single healthcare trust) retrospective case-controlled study approved by the Southern Health and Social Care Trust (SHSCT) (audit department approval January 2024). The inclusion criteria are as follows: (1) patients who were admitted to the SHSCT surgical service with an episode of ALGB; (2) cross-sectional imaging (CSI) during their index admission was required; and (3) patients aged 65 or older. CSI in this case could be any modality that allowed for psoas muscle analysis at the level of L3. Clinical coding provided the patient list for inclusion in the study. In total, 105 patients underwent CSI after an episode of ALGB between January 2017 and June 2022. Two patients were excluded due to an inability to evaluate PMI on CT imaging due to the presence of retroperitoneal pathology. 

PMI

**Figure 1:**
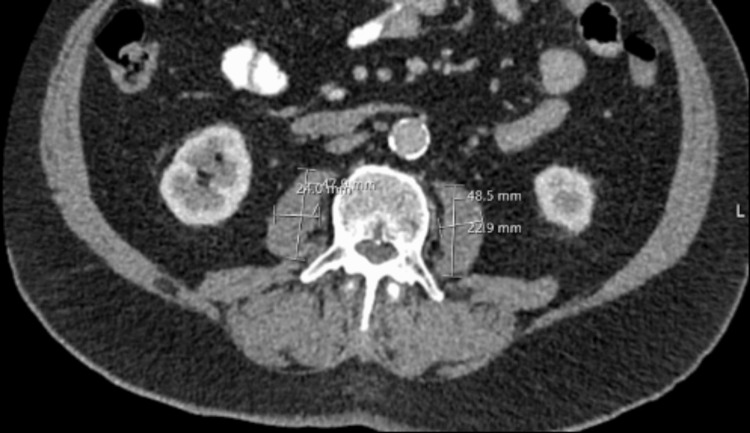
Image at the L3 vertebra. The measuring lines show the anteroposterior and transverse measurements used to calculate TPA. This was done using the picture archiving communications system (PACS) TPA: total psoas area

Patients that were included in the study had their total psoas area (TPA) measured by a radiology resident doctor (post-Fellowship of the Royal College of Radiologists (FRCR)) and then validated by a consultant radiologist (Figure 1). Picture archiving and communication system (PACS) was used for imaging access and analysis. TPA was calculated by virtually measuring the volume of psoas muscle on both the right and left sides in mm^2^ in axial slicing at the level of L3. Once TPA was calculated, patient height was obtained using a combination of data searching on the Northern Ireland Electronic Care Record (NIECR) and obtaining the patient’s physical care record. This allowed standardisation of the PMI for height giving PMI in mm^2^/m^2^.

Outcomes 

Demographic data was collected including age, sex, and height. To assess the overall impact of sarcopaenia, data was collected relating to mortality and length of hospital stay (LOHS). To analyse the level of frailty, patient’s discharge destination, readmission rate, and albumin levels were reviewed, the latter acting as a marker of overall nutritional status.

Statistical analysis 

A Chi-squared test assessed the association between sarcopaenia status and mortality. Calculated odds ratio (OR) with 95% confidence interval (CI) were used to quantify the strength and precision of the relationship between sarcopaenia and the odds of mortality. Kaplan-Meier survival curves were used to estimate the survival probabilities over time. Log-rank tests were conducted to compare the survival distributions between groups. Additionally, independent two-sample t-tests were performed to compare continuous variables such as albumin levels and LOHS between patients with and without sarcopaenia.

All statistical analyses were performed using Python version 3.9 (Python Software Foundation; Wilmington, Delaware, USA) with usage of the following libraries: Pandas (The Pandas Development Team), NumPy (Harris et al., 2020), SciPy (Virtanen et al., 2020), Statsmodels (Seabold and Perktold, 2010), and lifelines package (Davidson-Pilon, 2019).

## Results

A total of 105 patients, after an episode of ALGB, having undergone CSI in the SHSCT during the study dates were analysed. Two patients had to be excluded due to retroperitoneal pathology that did not allow for analysis of TPA at the level of L3. Of the 103 patients, 48 were males and 55 were females. Demographic data is summarised in Table 1. 

**Table 1:** Table illustrating gender-specific demographic data PMI: psoas muscle index; IQR: interquartile range

Category	Male	Female
Number of patients	48	55
Median PMI mm²/m² (IQR)	489.0 (392.0-541.0)	383.0 (364.0-414.0)
-Sarcopaenia	409.98 (378.48-485.33)	316.28 (302.27-343.55)
-Non-sarcopaenia	662.34 (603.99-755.01)	530.45 (489.79-617.05)
Median survival time days (IQR)	622.0 (478.0-818.0)	698.0 (506.0-829.0)
Median age at episode (IQR)	75.0 (70.0-81.0)	75.0 (70.0-79.0)
Sarcopaenic patients (n, %)	20 (41.67%)	12 (21.82%)
Median albumin levels (mg/dl) (IQR)	37.5 (32.0-40.2)	39.0 (36.5-43.0)
Median hospital stay (days) (IQR)	6.0 (3.0-10.0)	5.0 (3.0-7.0)

**Figure 2:**
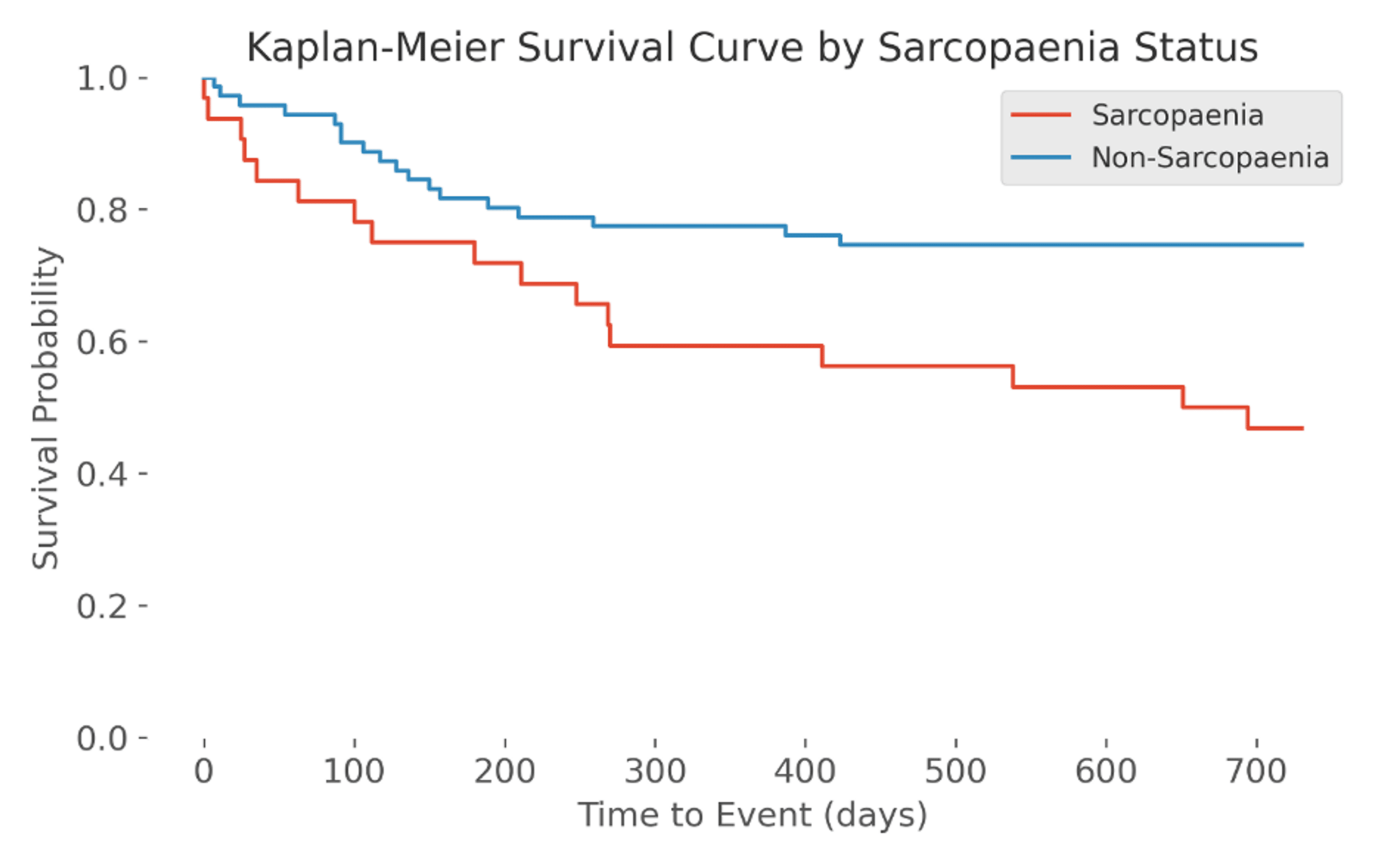
Kaplan-Meier analysis in sarcopaenia compared with non-sarcopaenia without gender stratification

**Figure 3:**
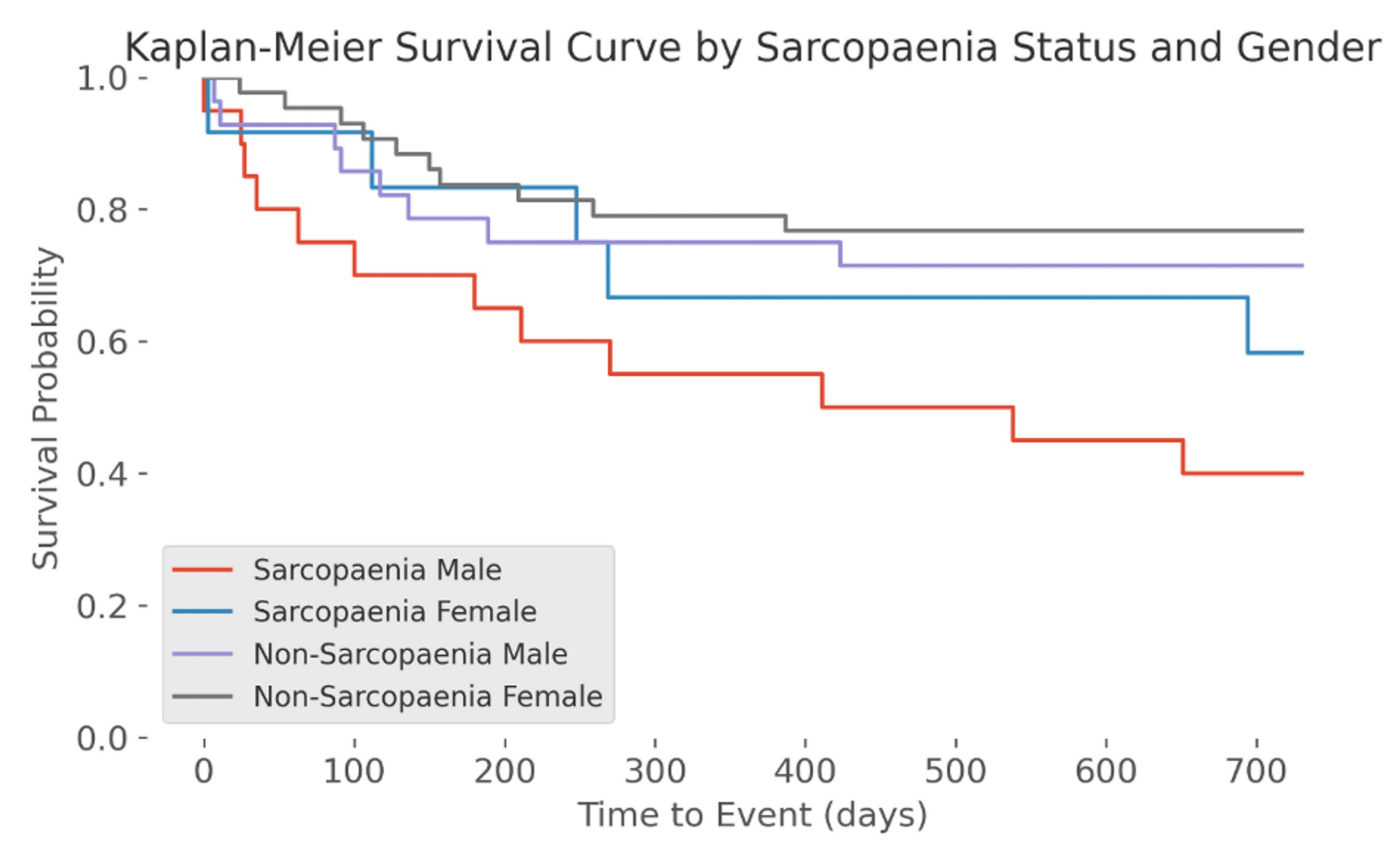
Kaplan-Meier analysis in sarcopaenia compared with non-sarcopaenia with gender stratification

The study included 32 sarcopaenic patients and 71 non-sarcopaenic patients. A total of 53% (n = 17) of sarcopaenic individuals had died within two years of an episode of ALGB compared to 25% (n = 18) in the non-sarcopaenic group. There was a statistically significant difference in mortality between sarcopaenic and non-sarcopaenic groups (Chi-square: 1, N = 103 = 7.582, p = 0.005888; OR: 3.34; 95% CI: 1.41-7.91). The Kaplan-Meier survival analysis is shown in Figure 2. Using log-rank test, a significantly lower survival probability in the sarcopaenic group (p < 0.001) was seen. There was also a statistically significant reduction in survival probability in both male (p < 0.001) and female (p < 0.001) sarcopaenic groups (Figure 3).

**Figure 4:**
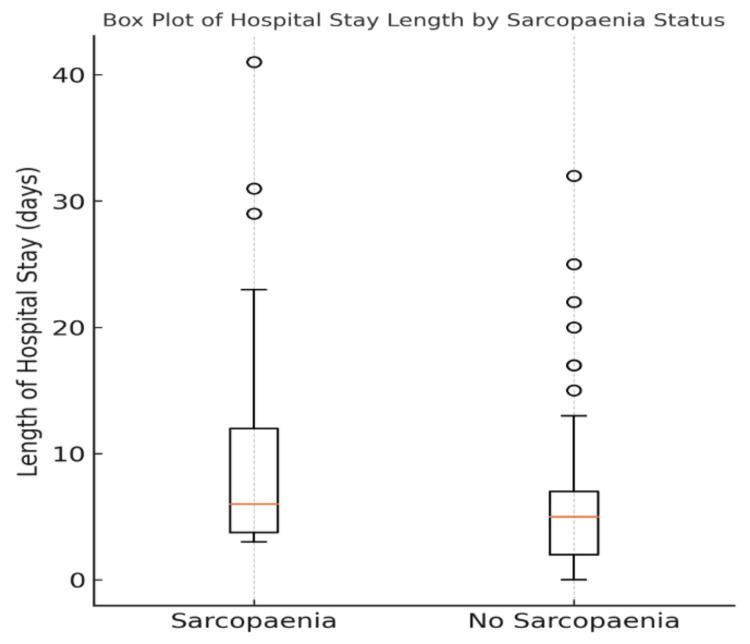
Box plot showing median hospital stay and IQR. This shows the longer median hospital stay for sarcopaenic patients in comparison to non-sarcopaenic patients

**Figure 5:**
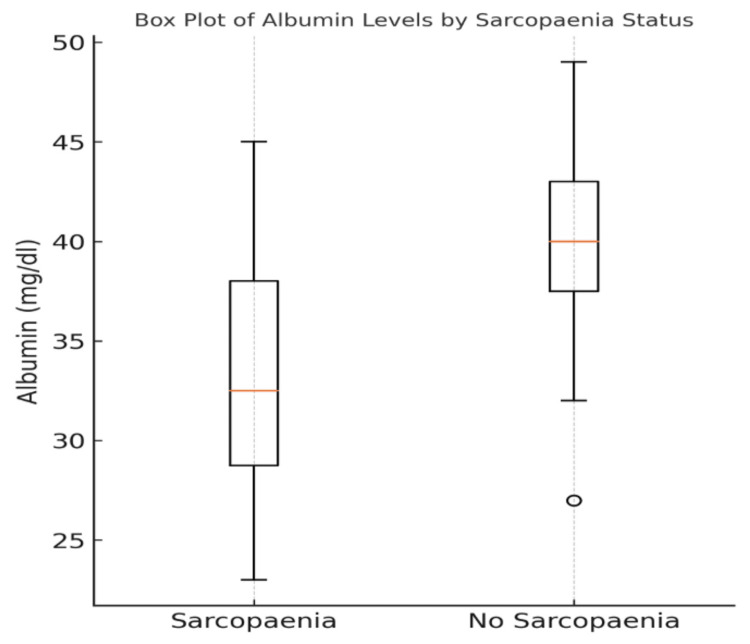
Box plot showing the mean albumin levels and 95% confidence intervals. This shows a significant (p < 0.001) decrease in albumin levels in sarcopaenic patients in comparison to non-sarcopaenic patients

Further analysis was performed in these patients to ascertain the extent of frailty and the effect of sarcopaenia. There was a statistically significant difference in LOHS. The median hospital stay for sarcopaenic individuals was six days (3.75-12.5 days), while non-sarcopaenic patients had a lower median hospital stay of five days (2-7 days). The Mann-Whitney U test shows a statistically significant association (p = 0.038) (Figure 4). There was no statistically significant association in discharge destination (OR: 1.95; 95% CI: 0.68-5.61; p = 0.1072). An increased LOHS was associated with patients discharged to additional care settings (p = 0.017). Albumin levels were analysed as a marker of overall nutrition (Figure 5). There was a significant difference in the mean albumin level in sarcopaenic patients (33.06 mg/dl ± 6.25 mg/dl) compared to non-sarcopaenic patients (40.14 mg/dl ± 4.49 mg/dl) (p < 0.001).

## Discussion

Multiple studies have examined the effect of sarcopaenia on postoperative outcomes following laparotomy [14,15,16]. There have also been studies analysing the effect of sarcopaenia on postoperative outcomes across various surgical specialties including vascular surgery, orthopaedic surgery, and neurosurgery. To our knowledge, this is the first study that examines the association between sarcopaenia and surgical pathology that is usually managed conservatively. 

The gold standard for the assessment of sarcopaenia is the CSI [12,17]. In the context of the study, we used human expertise to measure TPA, which allowed the calculation of the PMI. This was carried out by a radiology resident doctor in conjunction with a consultant radiologist who validated the results. There are various software packages that are available to calculate the TPA. Previous studies investigating sarcopaenia have used software packages such as IMAGEJ ( National Institutes of Health, Bethesda, Maryland, USA), Slice-O-Matic (TomoVision, Montreal, Quebec, Canada), and the UltraVisual software package (Merge Emageon, Birmingham, Alabama, USA). A study by Jones et al. suggested that these packages do not show a difference in accuracy when compared to the methods used in this study. There are other steps required to generate TPA, such as image anonymisation, software training, as well as the overall cost associated [18]. Therefore, the measurement of TPA at L3 by radiologists has given an accurate and reproducible way to assess sarcopaenia when imaging is available. 

Our primary outcome measure was the mortality risk following an episode of ALGB. The results of this study suggest that CSI-defined sarcopaenia at L3 is associated with increased all-cause mortality following an episode of ALGB. Our data suggests a 3.34 times increased likelihood of death following an episode of ALGB within two years in sarcopaenic patients (p = 0.005888). The Kaplan-Meier analysis showed an increased probability of mortality in sarcopaenic patients, over time. This is the case for males and females independently. A study by Lattanzi et al. examined the effect of sarcopaenia on bleeding and indicated that the rate of refractory bleeding secondary to varices is higher in sarcopaenic patients [19]. It is one of a few studies that has examined adverse outcomes in sarcopaenia following an episode of bleeding. It is important to note, however, that their primary outcome measure was not mortality, further emphasising the lack of research in this area. 

The secondary outcome measures in this study are related to the effect of sarcopaenia on non-mortality outcomes. Frailty is an important consideration in the analysis of surgical patients and their fitness. A study by Tan et al. suggested that frail patients were four times more likely to suffer major complications following colorectal surgery [20]. As such, identifying frailty early and taking steps to identify patients at risk is crucial. Sarcopaenia is a recognised element of frailty and ultimately is indicative of a patient’s ability to return to their functional baseline following hospital admission [21]. To evaluate the effect of sarcopaenia, we examined patients' LOHS, discharge destination, and albumin levels. In sarcopaenic patients, the median LOHS was higher than in the non-sarcopaenic group (p = 0.038). Studies have repeatedly shown that patients are more likely to have an increased LOHS when they are sarcopaenic. This tends to be more pronounced following operative intervention [13]. Increased LOHS tends to attenuate muscle atrophy which leads to worsening sarcopaenia and ultimately increasing frailty [22].

Generally, patients with sarcopaenia are less likely to be discharged to their own home and would require an enhanced provision of care [23]. Within our patient cohort, there was no statistically significant association between sarcopaenia and requiring increased levels of care on discharge. There was, however, a significant increase in LOHS in patients awaiting additional care arrangements. Sarcopaenic patients are more likely to have complex care needs. These needs are less likely to be met during hospitalisation, leading to an exacerbation of frailty [24].

An important element of sarcopaenia is thought to be malnutrition. Albumin is one biomarker useful in determining overall nutritional status [25]. In our patient cohort, there were lower albumin levels in patients with sarcopaenia, indicating an association. As such, we suggest that sarcopaenic patients should have regular input from a dietician to optimise their nutritional intake during admission. This emphasises the role of the multidisciplinary team (MDT) in managing these frail, likely comorbid patients. 

There are several limitations to the study. Firstly, the data was collected retrospectively which can lead to issues pertaining to recall bias and selection bias. Secondly, we had a relatively small number of patients included in the study (n = 103) and a relatively short follow-up period of two years. Thirdly, the assessment of muscle mass did not take into account overall body habitus; although the TPA was standardised for height, we did not include the patient’s body mass index (BMI). Additionally, we did not have access to clinical frailty scores. 

## Conclusions

Sarcopaenia is an important risk factor for all-cause mortality in surgical patients following ALGB. The easiest way to assess this is by way of CSI. Although radiation risk and indication for CT scanning may limit its use, CSI should be considered where resources permit.
